# Epstein-Barr virus BART microRNAs in EBV- associated Hodgkin lymphoma and gastric cancer

**DOI:** 10.1186/s13027-020-00307-6

**Published:** 2020-06-23

**Authors:** Valli De Re, Laura Caggiari, Mariangela De Zorzi, Valentina Fanotto, Gianmaria Miolo, Fabio Puglisi, Renato Cannizzaro, Vincenzo Canzonieri, Agostino Steffan, Piero Farruggia, Egesta Lopci, Emanuele S. G. d’Amore, Roberta Burnelli, Lara Mussolin, Maurizio Mascarin

**Affiliations:** 1 Immunopathology and Cancer Biomarkers, Department of Translational Research, Centro di Riferimento Oncologico di Aviano (CRO), IRCCS, Aviano, PN Italy; 2 Medical Oncology and Cancer Prevention, Department of Medical Oncology, IRCCS, Centro di Riferimento Oncologico di Aviano (CRO), Aviano, PN Italy; 3 Department of Medicine, University of Udine, Udine, Italy; 4 Gastroenterology, Department of Medical Oncology, Centro di Riferimento Oncologico di Aviano (CRO), IRCCS, Aviano, PN Italy; 5 Pathology, Department of Translational Research, Centro di Riferimento Oncologico di Aviano (CRO), Aviano, PN Italy; 6 Department of Medical, Surgical and Health Sciences, University of Trieste Medical School, Trieste, Italy; 7 Pediatric Hematology and Oncology Unit, Oncology, Department, A.R.N.A.S. Ospedali Civico Di Cristina e Benfratelli, Palermo, PN Italy; 8 Nuclear Medicine Department, Humanitas Clinical and Research Hospital, Via Manzoni 56, 20089 Rozzano, MI Italy; 9 Department of Pathology, San Bortolo Hospital, Vicenza, VI Italy; 10 Pediatric Hematology-Oncology, Azienda Ospedaliera Universitaria, Ospedale Sant’Anna, Ferrara, FE Italy; 11 Pediatric Hemato-Oncology Clinic, Department of Women’s and Children’s Health, University of Padua, Institute of Paediatric Research Fondazione Città della Speranza, Padua, PD Italy; 12 Pediatric Radiotherapy Unit, Centro di Riferimento Oncologico di Aviano (CRO), IRCCS, Aviano, PN Italy

**Keywords:** EBV, Epstein-Barr virus; miRNA, Micro RNA; BART, BamHI fragment a rightward transcript, HL, Hodgkin lymphoma; GC, Gastric carcinoma cancer

## Abstract

**Background:**

EBV produces miRNAs with important functions in cancer growth, tumor invasion and host immune surveillance. The discovery of EBV miR-BARTs is recent, and most of their functions are still unknown. Nonetheless, some new studies underline their key roles in EBV-associated malignancies.

**Main body:**

In EBV-associated tumors, the expression profile of miR-BARTs varies according to the cell type, autophagic process and signals received from the tumor microenvironment. By the same way of interest is the interaction between tumor cells and the tumor environment by the release of selected EBV miR-BARTs in addition to the tumor proteins trough tumor exosomes.

**Conclusion:**

In this review, we discuss new findings regarding EBV miR-BARTs in Hodgkin lymphoma and gastric cancer. The recent discovery that miRNAs are released by exosomes, including miR-BARTs, highlights the importance of tumor and microenvironment interplay with more specific effects on the host immune response.

## Background

Epstein-Barr virus (EBV) is a double-stranded DNA virus that replicates in the human oral epithelium (lytic phase) and is transmitted by saliva. Following the primary infection, EBV persists as a latent infection in B cells and epithelial cells. About 200,000 cancer cases per year are attributed to EBV worldwide [1]. EBV is associated with many benign and malignant lymphoproliferative disorders of B and T lymphocytes and is particularly frequent in lymphomas related to congenital and iatrogenic immunodeficiencies. In addition it is sometimes associated with carcinomas (nasopharyngeal undifferentiated carcinoma, gastric carcinoma and occasionally epithelial tumors of the lung, breast and salivary gland) and sarcoma (Inflammatory EBV-positive follicular dendritic cell sarcomas and subgroup of leiomyosarcomas in immunodeficient individuals, particularly HIV positive).

The virus enters cells by different routes. B cells are infected when the EBV envelope glycoprotein gp350 binds the Complement receptor type 2 (CR2/CD21) and the entire virus is taken up by endocytosis [2]. Epithelial cells are infected mainly by direct fusion at the cell surface where several proteins act as cofactors (e.g. ephrin receptor A2 [3], integrins [4] and nonmuscle myosin heavy chain IIA [5]; then the viral EBV capsid is released into the cell. In addition, the virus can be transmitted from B cells to epithelial cells and vice versa through the lytic and latent infection cycles.

The role of EBV in oncogenesis is only partially known. Multiple factors, including the EBV genome and environmental and host genetic factors, are necessary for oncogenesis. Viruses contribute to the development of human tumors by inducing cell proliferation through the abnormal activation of oncogenes or the silencing of tumor suppressors. In addition, EBV suppresses the host immune system. Virus reactivation, due to a decrease in host immune status (e.g. HIV infection, organ transplant), may favor the occurrence of EBV-associated lymphoproliferative malignancies.

During lytic infection all viral genes are expressed, while during latent infection a limited number of viral genes is expressed. The viral expression profile varies according to tumor cell type (Table 1).

**Table 1:** Protein patterns distinguishing different EBV states

Cell type	EBV state	BZLF1	BARF-1^a^	miR-	miR-	EBNA proteins						**LMP**			
				BARTs	BHRF1s	1	2	3A	3B	3C	LP	1	2A	2B	EBER
Plasma cell	Lytic	+	+	+	+#	–	–	–	–	–	–	–	–	–	+
PEL, NPC															
Memory B cell	Latency 1	–	–	+	−/+	+	–	–	–	–	–	–	–	–	+
Germinal center B	Latency 2	–	–	+	−/+	+	–	–	–	–	+	+	+	+	+
cell, HD, NPC, BL															
Naïve B cell, LCBL	Latency 3	–	–	+	+	+	+	+	+	+	+	+	+	+	+
Gastric carcinoma	Latency 1–2	–	+	+	–	+	–	–	–	–	+	–	+	+	+

^a^ BARF1 promoter is cell-type specific: in viral latency, BARF1 is exclusively expressed in epithelial tumors such as nasopharyngeal and gastric carcinoma but not in lymphoma. # miR-BHRF1s are restricted to lymphoblastoid cell lines (LCL) and nasal NK/T cell lymphoma. BARF and EBER, RNA; *BL* Burkitt lymphoma, *DLCBL* Diffuse large B-cell lymphoma; *EBNA* EBV nuclear antigen; *HD* Hodgkin disease; *LMP* Latent membrane proteins; *NPC* Nasopharyngeal carcinoma; *PEL* Reactivity of peripheral blood lymphocytes

**Fig. 1:**
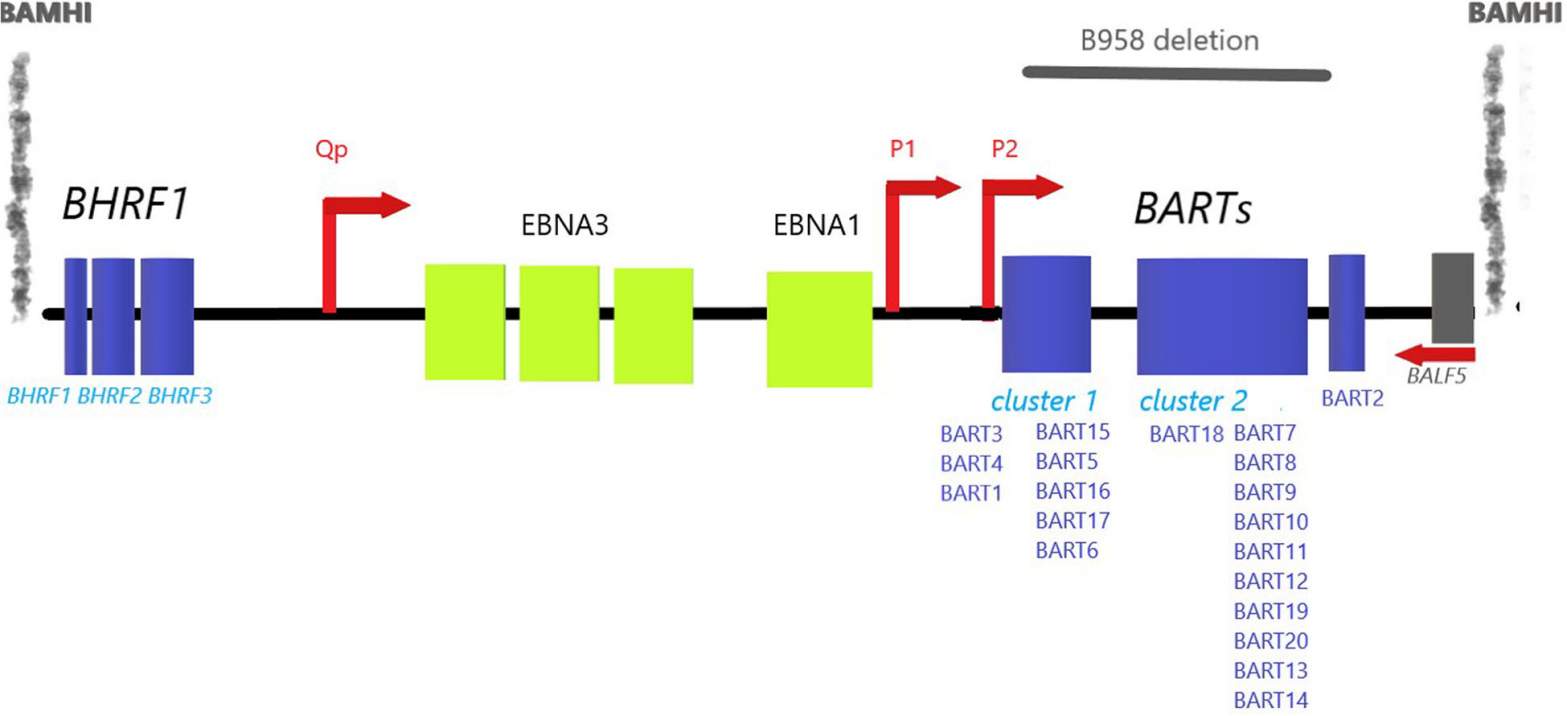
Genomic map of the EBV region containing the BAMHI-I fragment. miR-BamHI fragment H rightward open reading frame 1 (BHFR1), latent EBV-encoded nuclear antigen 3 (EBNA3), latent EBV-encoded nuclear antigen 1 (EBNA1), miR-BamHI A rightward transcripts (BARTs) in sequence order. Genomic deletion in the EBV B958.9 strain is shown. P1, promoter 1; P2, promoter 2; Qp, Q promoter are indicated by arrows

In the latent form, EBV infection does not produce virions and the episome is located in the nucleus of tumor cells [6]. The latent form is favorable for EBV persistence since, by reducing viral gene expression and the expression of antigens, it helps to elude immune system. The expression of non-coding RNAs mediates immune escape rather than cellular transformation. Persistence of the viral episome requires production of the EBV nuclear antigen 1 (EBNA1) from the origin of plasmid replication Bam H1Q promoter (Fig. 1). Lytic EBV replication was thought to destroy latently infected cells and thereby inhibit tumorigenesis. However, recent studies found that initiation of the lytic cycle may also support EBV-driven malignancies. Recently, a new EBV state, intermediate between the lytic and latent forms, has been described; in this state, the expression of some lytic genes does not result in the production of effective virions [7]. The intermediate state of EBV originates from intragenic deletions that frequently occur in the BART region or in essential lytic genes (Fig. 1). The mechanisms underlying BART deletions are poorly understood. One hypothesis is that certain miR-BARTs target viral lytic proteins (e.g. BZLF1 and BRLF1) and thereby repress reactivation from latency. Wild-type EBV occasionally executes full lytic replication, leading to progeny production, but such cells are destined to die or be eliminated by the immune system. It is assumed that the intermediate state is advantageous for the virus to block reinfection of the same cells, although producing proteins and RNAs resulted in leaky expression of viral lytic genes but consented cell survival.

### MicroRNAs

MicroRNAs (miRNAs) are short non-coding single-stranded RNA molecules implicated in the post- transcriptional regulation of genes via either translation repression or RNA degradation. EBV is the first human virus shown to encode miRNAs. EBV produces 25 miRNA precursors, which contain 49 mature miRNAs. EBV miRNAs are all overexpressed during latency [8]. EBV miRNAs can be transferred by secreted exosomes from infected cells. Thus, these miRNAs are potential factors for genome regulation of both infected and uninfected cells that sustain the tumor microenvironment.

miRNA precursors are clustered in two regions of the EBV genome (Fig. 1). The BamHI fragment H rightward open reading frame 1 (BHRF1) gene encodes three miRNA precursors (BHRF1 to 3) that generate four mature miRNAs. The BamHI fragment A rightward transcript (BART) region contains 22 miRNA precursors (BART1 to 22) that produce 44 mature miRNAs [9]. The BART region is further subdivided into subclusters 1 and 2, with the miRNAs ebv-miR-BART2-5p and ebv-miR- BART2-3p located downstream of these two clusters. Distinct EBV miRNA profiles play a crucial role in cancer by manipulating host cells. Thus, they have been proposed as markers of distinct EBV-associated tumor types and of poor prognosis.

### BART miRNAs and lnc

Regulatory RNAs are distinguished, by sequence length, into small regulatory RNAs (< 100 nucleotides) and long noncoding (lnc) RNAs (> 200 nucleotides). Both miR-BARTs and lnc-BARTs are expressed at high levels in EBV+ malignancies, suggesting they have a role in tumorigenesis [10]. miR-BARTs have been mostly studied in nasopharyngeal cancer (NPC). In this cancer, they contribute to virus latency, cell proliferation and apoptosis, metastasis and tumor recurrence, and participate in the regulation of tumor cell metabolism and immune evasion [11]. Alternative splicing of BARTs results in multiple spliced forms of Lnc-BARTs, with putative open reading frames: BARF0, RK- BARF0, RPMS1, and A73 [12]. They did not produce proteins and are believed to regulate cell growth by modulating host gene expression. The mechanism is not yet elucidating. However, since lnc-BARTs localize in the nucleus, some evidence resulting from studies on BART RNAs in NPC, suggest that lnc-BARTs could mediate epigenetic regulation of gene expression by interacting with DNA methylation and the chromatin remodelling machinery [13]. Lnc-BARTs was found to control Polymerase (Pol II) at the promoter region and regulate interferon (IFN)-beta1 and chemokine-8 (CXCL8) expression in NPC [10]. However, continued investigations are still required to fully characterize the role of lnc-BARTs but it strongly possible that EBV adopt a strategy to express abundant levels of lnc-BARTs to suppress most antigenic latency EBV proteins, thus reducing host immune response in EBV-associated malignancies where only EBNA1 was found expressed.

In addition to the spliced BARTs, several EBV-encoded miR-BARTs are also encoded by the intronic regions of the BARTs. The biogenesis of miR-BARTs has been studied by Kim and Lee [14], who identified several promoters. The P1 promoter is the predominant start site. However, transcripts containing upstream sequences have been identified, suggesting that there are multiple start sites (Fig. 1). The promoter regions are possibly regulated by SP1, Ets transcription factor, Jun family members, IRF (interferon regulatory factor) proteins, c-myc, and C/EBP proteins. The transcription through exon 1 and protection from methylation around this region suggest that this region is the active template for miRNA sequences downstream of exon 1. A study has suggested that miR-BARTs are mainly transcribed by RNA polymerase II and undergo processing to be transported from the nucleus and cut at the state of pre-miRNAs by enzymes like RNAse III "Drosha" and endoribonuclease Dicer, similarly as happens for cellular miRNAs [14]. It is not yet known how BART promoters are regulated to maintain the high level of expression of BARTs seen in NPC and cancer.

Due to this important function, BARTs were proposed as biomarkers and targets of therapy in NPC. Here, we summarize the roles of the most important miR-BARTs, based on both their functions and their association with distinct EBV-associated malignancies, in Hodgkin lymphoma and gastric cancer.

### BART miRNAs in Hodgkin lymphoma

In Hodgkin's lymphoma, there are very few cancer cells, and these are immersed in a microenvironment particularly rich in immune cells, which constitute the inflammatory niche necessary for the tumor growth and survival. The prognostic significance of EBV in Hodgkin lymphoma is controversial, however some studies indicate that a poorer outcome may be related to the age of the patients and is worse in those over 50 years. Since in the elderly the immune response is less efficient than in young people, this observation had reinforced the importance of the host immune response in the control of EBV+ Hodgkin lymphoma [15, 16].

Hodgkin lymphoma, like NPC and NK/T cell lymphoma, shows a latency II type, which is characterized by the expression of EBNA-1, LMP-1, LMP-2A, and LMP-2B. While the EBV latent proteins have been investigated intensively in Hodgkin lymphoma, the roles of EBV miRNAs are just starting to be explored. Data so far indicate that EBV-associated tumors upregulate a subset of miR-BARTs that are silenced in latently normal infected cells in vivo [17]. It is not yet known how miR-BART expression levels are regulated. A possible mechanism is that the stability of miRNAs depends on the presence of their target mRNA in the cell.

Recently, lnc-BARTs expression was found to be regulated by NF-κB signaling in the induction of EBV lytic replication [18, 19]. Persistent NF-κB activation, is a hallmark of the malignant Hodgkin and Reed-sternberg (HRS) cells [20, 21]. The latent LMP1 gene of EBV, expressed in Hodgkin lymphoma, mimic a constitutive activation of the CD40 receptor and thereby actives NF-κB rescuing germinal center B-cell with inefficient immunoglobulin variable (IgV) sequences from apoptosis [22]. By the same way NF-κB activation leads to the production and release of a large quantity of pro-inflammatory cytokines in the tumor microenvironment and increases the lncBARTs expression (i.e mostly *RPMS1 lnc-*BART [19]) that sustain either HL growth and EBV latency.

A study that investigated miR-BARTs in Hodgkin lymphoma found the expression of miR-BARTs 19- 3p and 13-3p, but the overall miR-BART expression was low compared to other malignancies [23].

Plasma BART19-3p is the most significant miRNA marker for NPC diagnosis [24, 25]. It is highly expressed in tumors, with a latency III like the NK/T-cell lymphoma [26]. Today, the role of BART19-3p is not understood.

Of interest, recent studies showed that tumor cells, including EBV+ Hodgkin lymphoma cells, secrete exosomes [27, 28]. Exosomes from Hodgkin lymphoma are taken up by macrophages (CD68+ cells). Tumor-associated macrophages (TAMs) have an important role in the progression of Hodgkin lymphoma. They are a major immune component of the tumor microenvironment and a good prognostic biomarker for disease-free survival and overall survival. TAMs are involved in various aspects of tumor progression including aberrant cytokine secretion, a driver of immune checkpoint blockade particularly important in Hodgkin lymphoma where the PD-L1 gene is amplified in tumor cells [29]. TAMs are also important in tumor matrix remodeling, angiogenesis, and resistance to treatment [30].

Macrophages are versatile cells capable of adapting to the signals present in the tumor microenvironment leading to different states of activation (M1, M2 and M2-like) with distinct sets of surface receptors and effector molecules. M1 and M2 states depend on the main cellular sources of IFN-γ (M1, enhances microbial killing and increases cytokine production) and IL-4 (M2, fundamental to limit tissue damage caused by inflammation). There is an association between a high infiltration of M2 macrophages (CD163+) in Hodgkin lymphoma tissue and worse disease outcome.

Exosomes are composed of a lipid bilayer with a diameter of 50–200 nm and they transport biological molecules. Only exosomes derived from EBV-positive Akata cell line, but not those from EBV-positive B95-8 cell line having a deletion in BART cluster 2 region (Figure 1), caused severe lymphoproliferative diseases in a humanized mouse model [27]. These results emphasize the role of BARTs in lymphomagenesis. The levels of both M1 (CD68+) and M2 (CD163+) macrophages in the spleen of mice infected with Akata are higher than in mice B95-8-infected. Macrophages recognize phosphatidylserine, also known as the “eat-me” signal, exposed by both apoptotic bodies of lymphoma- derived exosomes and exosomes, and the uptake of miR- BARTs from exosomes causes macrophage phenotypic changes (e.g. increased survival and increased production of cytokines such as pro-inflammatory tumor necrosis factor (TNF)-α and immunosuppressive IL-10). Thus, miR-BART regulates gene expression in macrophages and directs macrophages towards a pro-tumor inflammatory state and a reduced direct host-defense against EBV.

miR-BART13-3p is the most frequent miR-BART in Hodgkin lymphoma, and it is released into the circulation via exosomes [23, 31]. In NPC, EBV-miR-BART13-3p enhanced the migration of tumor cells and caused metastasis by driving an epithelial-mesenchymal transition via downregulation of the tumor suppressor AB12 [32].

miR-BART13-3p has a superior diagnostic performance than anti-EBNA1 IgA serology and EBV DNA load for NPC compared to asymptomatic EBV-infection (i.e. healthy donors and non-NPC tumors, EBV-associated diseases) [31].

miR-BART2-5p, an anti-sense miRNA to EBV DNA polymerase BALF5, was found in EBV+ Hodgkin lymphoma cells and in macrophages [33]. This finding suggests that miR-BARTs, through exosomes, have a role in Hodgkin lymphoma. Notably, miR-BART2-5p inhibited B-cell receptor (BCR)-mediated NF-kB activation and was upregulated during the lytic phase [34]. As a result, the level of BALF5 protein was reduced as well as the amount of virus released from EBV-infected cells, suggesting that it protects latent cells from EBV reactivation [35, 36]. miR-BART2-5p is abundant in the circulation of patients with EBV+ nasal natural killer lymphoma and NPC, so in both these cancers it is a diagnostic and prognostic biomarker [32, 37]. miR-BART2- 5p targets MHC class I polypeptide-related sequence B (MICB) to evade recognition and attack by NK cells [38]. MICA and MICB are ligands of the NKG2D receptor, which plays an important role in the immune response by activating NK and T cells. NKG2D was detected in about 38% of NK-cell neoplasms and cytotoxic T-lymphocyte-derived lymphomas [39]. MIC expression is restricted to endothelial cells, fibroblasts, epithelial cells, and tumor cells. Since NK cells efficiently recognize and kill tumor cells bearing NKG2D ligands, a reduction of MICB on the tumor surface potentially inhibits the “danger signals” that alerts the innate immune system, and therefore reduces tumor elimination.

### BART miRNAs in gastric cancer

EBV+ gastric cancer represents about 10% of all gastric cancer cases. EBV+ gastric cancer predominates in young men and localizes mainly in the proximal region of the stomach [40]. It is molecularly characterized by extreme CpG hypermethylation, frequent mutations in PIK3CA and ARID1A, lack of TP53 mutations [41, 42], and overexpression of IFN-□ [43] and PD-L1 [44].

Deregulation of the NF-κB signaling cascade is also view as a pro-inflammatory-pathway that induce carcinogenesis, either in lymphoid cells (e.g. Hodgkin lymphoma) and in epithelial tumors (e.g. gastric cancer) [45].

In EBV-infected GC, 99% of all virally derived polyadenylated transcripts are from BARTs, resulting in the production of miR-BARTs and lnc-BARTs [46]. They were more abundant in epithelial tumor cells than in lymphoid cells [47].

EBV latent form expressing EBER, EBNA1 and miR-BARTs has been detected in EBV+ gastric cancer [47]. Latent protein LMP2A has also been detected in some cases, while LMP1 and LMP2 are often absent in non-invasive EBV+ gastric cancer [48, 49]. Almost all EBV+ gastric cancers express high levels of nearly all miR-BARTs, while NPC and GC did not express miR-BHRF1 [50]. EBV miR-BARTs have been reported to regulate the expression of viral genes BZLF1 and BRLF1, to reduce both the lytic replication of the virus [51] and the oncogenic latent protein LMP1 [52].

Therefore, they regulate the viral cycle and the oncogenic transformation of gastric cells. Of note, miR-BARTs are highly expressed (>10% of the total pool of miRNAs) in EBV+ gastric tumor cells [53]. However, it is important to pay attention to the effects of the tumor milieu, which produces potent inflammatory mediators such as IFN-γ that upregulate the transcription of mRNA targets, leading to differences in expression of mRNAs between gastric cancer cell lines and tissue samples [54]. EBV-positive gastric cancer tissue samples had intact MHC-I antigen presentation, including EBV antigen presentation, and increased T-cell infiltration than EBV-negative gastric cancer tumor samples [54]. These findings may explain the better outcome of patients with EBV+ gastric cancer than with other types of this tumor [55].

The miR-BART expression profiles of clinical samples of EBV+ gastric cancer have been reported [56]. In that study, miR-BART7-3p had the highest expression level, followed by miR-BART9-3p, miR-BART1-3p, miR-BART5-5p, miR-BART14-3p, miR-BART10-3p, miR-BART4-5p, miR-BART1-5p, miR-BART2-5p, and miR-BART15. Low expression was reported for miR-BART2-3p, miR-BART7-5p, miR-BART9-5p, miR-BART10-5p, miR-BART14-5p, miR-BART18-3p, miR-BART20-5p, and miR-BART20-3p. According to an in-silico analysis, this expression profile associated with oncogenesis, cell adhesion, signal transduction, and apoptosis, all of which are essential for the development and progression of gastric cancer. However, since the discovery of miR-BARTs is relatively recent, the functions of most miR-BARTs in gastric cancer pathogenesis are only beginning to emerge. A list of these miRNAs with their main functions are reported in Table 2.

**Table 2:** miR-BART targets and their functions in different cancers

miR-BART	Target	Function	Disease	Reference
miR-BART13-3p	Tumor suppressor AB12	EMT	HL, NPC	[32]
mR-BART19-3p	Unknown	–	HL, NPC, ENKL	[23]
miR-BART2-5P	MICB BALF5	Ligand for activator NKG2D NK receptor RNA polymerase II and B-cell receptor NFKB activation	GC, NPC HL, ENKL	[34–38]
miR-BART7-3p	phosphatase and tensin homolog (PTEN) mothers against decapentaplegic homolog 7 (SMAD7)	Tumor suppressor, pro- inflammatory, stem-like	GC	[56–58]
miR-BART4-5p	BH3 interacting-domain death agonist (Bid)	Pro-apoptotic protein	GC	[56]
miR-BART17-5p	DNA transcription factor Kruppel-like factor 2 (KLF2)	Energy metabolism inflammation	GC	[59, 60]
miR-BART I,II cluster	bcl2-interacting mediators of cell death (Bim)	Pro-apoptotic protein Autophagy	GC	[61]
miR-BART3-3p	TP53	tumor suppressor	GC	[62]
miR-BART5- 3p	TP53 PIAS3	tumor suppressor inhibitor of pSTAT and pDL-1	GC	[44, 63]
miR-BART15-5p	NLRP3 inflammasome BRUCE and TAX1BP1	pro-inflammatory cytokines such as IL-1β and IL-18, gasdermin D- mediated pyroptotic cell death apoptosis	GC	[64–66]

*EMT* Epithelial-mesenchymal transition; *ENKL* Nasal NK7T-cell lymphoma; *G*C Gastric cancer; *HL* Hodgkin lymphoma; *NPC* Nasopharyngeal carcinoma

The low apoptotic status of EBV-positive gastric cancer tissue, compared to EBV-negative tissue, has been associated with the effect of miR-BART4-5p on the expression of the pro-apoptotic BH3 interacting-domain death agonist (Bid) [56]. The function of the pro-apoptotic Bid gene is to mediate, by forming heterodimers with either the agonist Bax or the antagonist Bcl-2, the mitochondrial damage leading to apoptosis.

miR-BART7-3p has been hypothesized to play a role in tumor growth and invasion through the suppression of phosphatase and tensin homolog (PTEN) protein and others against decapentaplegic homolog 7 (SMAD7) [57, 58]. PTEN is one of the main tumor suppressor genes involved in the regulation of the cell cycle. SMAD7 is a transforming growth factor beta (TGFβ) type 1 receptor antagonist. The SMAD7/ TGFβ interaction blocks IL-1R/TLR signaling, which subsequently reduces the expression of pro-inflammatory genes. SMAD7 production is induced by TGFβ, but also by epidermal growth factor, IFN-γ and TNF-α. Therefore, SMAD7 may also prevent metastasis in EBV+ gastric cancer. Moreover, by inducing a cancer stem cell-like alteration, SMAD7 enables cells to better resist chemoradiotherapy. Gold nanoparticles carrying an anti-miR-BART7- 3p antibody reduced tumor growth in an animal model, suggesting that this miRNA has a crucial role in epithelial cancer [67].

miR-BART17-5p is expressed in gastric cancer tissue [59, 60] and in EBV+ gastric cancer cell lines [60]. High miR-BART17-5p serum levels were found in NPC patients in association with progression and recurrence of the tumor [68–70]. In NPC, LMP1 is the main target of miR-BART17-5p [71]. However, since LMP1 expression is abrogated in EBV-positive gastric cancer, the main effect of miR-BART17-5p occurs on the transcription factor Kruppel-like factor 2 (KLF2) [59]. KLF2 negatively regulates energy metabolism of tumor cells and inflammation; thus, miRBART action on KLF2 positively favors gastric cancer cell migration and anchorage-independent growth.

Additionally, several miR-BARTs from clusters I and II were found to downregulate the expression of bcl2-interacting mediators of cell death (BIM) [61]. A previous study reported an association between reduction of BIM and progression of gastric cancer [72]. BIM was found to play a key role in gastric cancer by reducing autophagy, an adaptive response to apoptosis. Autophagy is a common response to starvation, which prolongs cell survival through the degradation and recycling of cellular macromolecules to provide energy and molecular precursors [73]. Other miR- BARTs (mainly miR-BART3-3p, miR-BART5-5p and miR-BART2-5p) were encoded by EBV to support host immune escape of gastric tumor cells.

In gastric cancer, miR-BART3-3p was found to inhibit tumor infiltration by NK cells and macrophages, by altering the senescence (temporary or permanent cell cycle arrest)-associated secretory phenotype (SASP) [62]. It seems that miR-BART3-3p directly targets the tumor suppressor gene TP53 and the TP53 target, p21, to produce this effect.

miR-BART5-5p also targets TP53, and thus promotes cell survival [74]. Of interest, it also directly targets PIAS3, an inhibitor of the activated pSTAT3 protein [59]. pSTAT3 migrates to the nucleus where it induces the transcription of several genes in gastric cancer. Since the PD-L1 gene promoter contains sequences for STAT3 binding, the result is of interest because gastric cancers EBV-positive and those having a high microsatellite instability are the best tumor candidate for immunological therapies (i.e. immune PD-1/PDL-1 checkpoint blockade) [44].

Another way to reduce immune surveillance is to release some miR-BARTs through exosomes.

Some miR-BARTs were found to be highly enriched in exosomes of EBV+ gastric cancer cell lines [75]. It is important to note that only some miR-BARTs are released in exosomes [76]. Several ways for miRNAs to enter vesicles have been hypothesized [77, 78]. Among the frequent miR-BART in exosomes of gastric cancer is miR-BART15-5p. This miR-BART regulates NLRP3 (NOD). NLRP3 is an intracellular sensor of danger that activates the NLRP3 inflammasome, leading to the release of pro-inflammatory cytokines (e.g. IL-1β and IL-18) and to gasdermin D-mediated pyroptotic cell death, which actives the innate immune response [65, 66]. Therefore, miBART15-5p limits inflammation to promote EBV infection [78]. Moreover, miR-BART15-5p may also have a role in the induction of apoptosis by targeting two inhibitory proteins of apoptosis, BRUCE and TAX1BP1 [64]. Therefore, it leads to death of tumor cells but also of cells around the tumor, including immune cells, through the release of exosomes.

## Conclusions

Interactions between EBV miR-BARTs and Hodgkin lymphoma and gastric cancer are still poorly understood. Nonetheless, in recent years, the importance of these molecules in cancer has been acknowledged. Of special interest is the regulation of their expression in different EBV-related malignancies. Some miR-BARTs are released through exosomes into the tumor microenvironment. The mechanism to sustain miR-BART expression and release in exosomes is unclear.

Hypermethylation is not involved. The best hypothesis is that they are regulated by RNA targets in the tumor cells. Modification of the tumor microenvironment through miR-BARTs transported by exosomes is important mainly for the effect on the host immune response and a better understanding of these biological mechanisms may be helpful for the development of innovative therapies. miR-BARTs are released by tumor cells into blood in a stable form, and some of them are abundant in EBV-associated malignancies. Some miR-BARTs have already been found to be useful biomarkers for the diagnosis of the first occurrence or recurrence of a malignancy. Several studies are currently investigating which miR-BARTs are associated with specific EBV+ malignancies and may have clinical utility. Altogether, this new information is important for the development of novel diagnostic and prognostic biomarkers and for identifying targets for the development of more effective therapies, including those that boost host immunity.

## Data Availability

“Not applicable”.

## Abbreviations

EBV: Epstein-barr virus
miRNA: Micro RNA
BART: BamHI fragment A rightward transcript
BHRF1: BamHI fragment H rightward open reading frame 1
HL: Hodgkin lymphoma
GC: Gastric carcinoma cancer
HIV: Human immunodeficiency virus
CR2/CD21: Complement receptor type 2
EBNA1: EBV nuclear antigen 1
Lnc: Long noncoding RNAs
NPC: Nasopharyngeal cancer
Pol: POLYMERASE
IFN: Interferon
CXCL8: Chemokine 8
IRF: Interferon regulatory factor
NK: Natural killer cell
LMP: Latent membrane protein
NF-κB: Nuclear factor-kB
TAMs: Tumor-associated macrophages
PD-L: Programmed death ligand
IL: Interleukin
IgV: Immunoglobulin variable
TNF: Tumor necrosis factor
MHC: Major histocompatibility complex
MICB: MHC class I polypeptide-related sequence B
PTEN: Phosphatase and tensin homolog
Bid: Pro-apoptotic BH3 interacting-domain death agonist
SMAD7: Decapentaplegic homolog 7
TGFβ: Transforming growth factor beta
KLF2: Transcription factor Kruppel-like factor 2
BIM: Bcl2-interacting mediators of cell death
STAT: Signal transducer and activator of transcription
NLRP3: NOD-, LRR- and pyrin domain-containing protein 3
EMT: Epithelial-mesenchymal transition
ENKL: Nasal NK7T-cell lymphoma
